# The Venom of the Spider *Selenocosmia Jiafu* Contains Various Neurotoxins Acting on Voltage-Gated Ion Channels in Rat Dorsal Root Ganglion Neurons

**DOI:** 10.3390/toxins6030988

**Published:** 2014-03-05

**Authors:** Zhaotun Hu, Xi Zhou, Jia Chen, Cheng Tang, Zhen Xiao, Dazhong Ying, Zhonghua Liu, Songping Liang

**Affiliations:** 1 Key Laboratory of Protein Chemistry and Developmental Biology of the Ministry of Education, College of Life Sciences, Hunan Normal University, Changsha, Hunan 410081, China; E-Mails: huzhaotun@163.com (Z.H.); zhouxi016@163.com (X.Z.); chenjia113322@163.com (J.C.); tangcheng1985ss@sina.com (C.T.); dazhongyin002@126.com (D.Y.); 2 Key Laboratory of Research and Utilization of Ethnomedicinal Plant Resources of Hunan Province, Department of Life Science, Huaihua College, Huaihua, Hunan 418008, China; E-Mail: 405542716@qq.com

**Keywords:** *Selenocosmia jiafu*, spider venom, RP-HPLC, MALDI-TOF-MS, patch clamp analysis, voltage-gated ion channel, DRG neuron

## Abstract

*Selenocosmia jiafu* is a medium-sized theraphosid spider and an attractive source of venom, because it can be bred in captivity and it produces large amounts of venom. We performed reversed-phase high-performance liquid chromatography (RP-HPLC) and matrix-assisted laser-desorption/ionization time-of-flight mass spectrometry (MALDI-TOF-MS) analyses and showed that *S*. *jiafu* venom contains hundreds of peptides with a predominant mass of 3000–4500 Da. Patch clamp analyses indicated that the venom could inhibit voltage-gated Na^+^, K^+^ and Ca^2+^ channels in rat dorsal root ganglion (DRG) neurons. The venom exhibited inhibitory effects on tetrodotoxin-resistant (TTX-R) Na^+ ^currents and T-type Ca^2+^ currents, suggesting the presence of antagonists to both channel types and providing a valuable tool for the investigation of these channels and for drug development. Intra-abdominal injection of the venom had severe toxic effects on cockroaches and caused death at higher concentrations. The LD_50 _was 84.24 μg/g of body weight in the cockroach. However, no visible symptoms or behavioral changes were detected after intraperitoneal injection of the venom into mice even at doses up to 10 mg/kg body weight. Our results provide a basis for further case-by-case investigations of peptide toxins from this venom.

## 1. Introduction

Spiders evolved approximately 400 million years ago from an arachnid ancestor [1]. Excluding insects, which are the primary prey of spiders, spiders are the most diverse and successful terrestrial invertebrates [2]. There are 43,244 described species in approximately 111 families, with an even greater number awaiting characterization [3]. Spiders are distributed all over the world and have conquered all ecological environments. Most spiders are relatively small (2–10 mm body length), although some large tarantulas may reach a body length of 80–90 mm. Many are specialized as snare builders (web spiders), whereas others actively hunt their prey (ground spiders or wandering spiders).

All spiders are predators and, with the exception of Uloboridae and Holarcheae, all spiders have a venom apparatus. The venom apparatus of spiders consists of a pair of chelicerae and venom glands. The shape and position of the venom gland differs among species. In the large “theraphosids”, the venom glands are quite small and lie inside the chelicerae. In the genus *Atypus*, the glands are composite, while in *Filistata* they are of a multilobular type, and in *Scytodes* they are bilobular [4].

**Figure 1:**
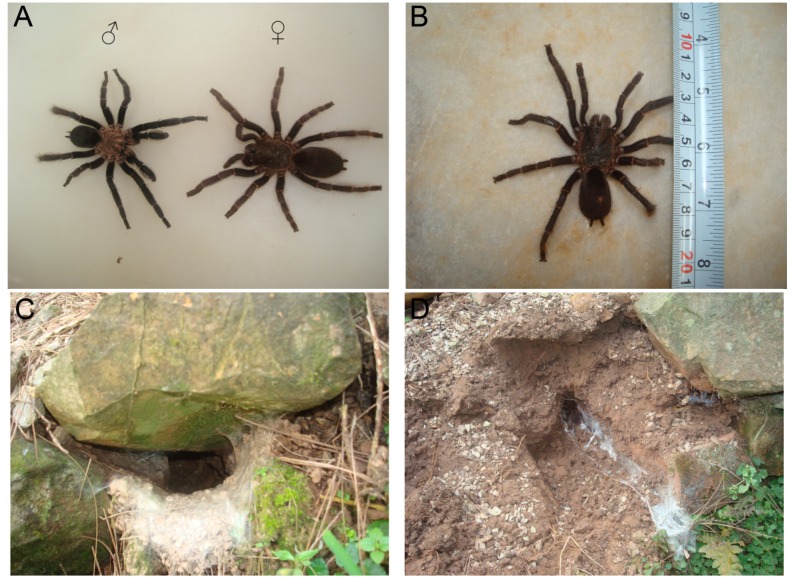
The spider *S*. *jiafu*and habitatburrow of the spider *S*. *jiafu*: (**A**) the spider *S*. *jiafu*; (**B**) A female *S*. *jiafu* has a body length of 50 mm and a leg span of 90 mm; (**C**) the burrow usually has a loose silken tube at the entrance; and (**D**) the construction of the burrow is horizontal or at a slight angle and the inner wall is covered with silk.

*S*. *jiafu* is one of the venomous spider species found in the hilly areas of Yunnan province and Guangxi province in the south of China. As a new species, *S*. *jiafu* was first discovered in Yunnan Province in 2008 [5]. The authors of this study found this spider specimen in 2012 in a hilly area of Ninming county in the Guangxi province. *S*. *jiafu* is a medium-bodied hairy spider (Figure 1A). The male and female *S*. *jiafu* spiders are described as follows: The male carapace is yellow-brown with a reticulated patch and dense fluff, and long hairs at its margins; yellow-brown chelicerae are covered with long brown hairs dorsally and yellow-brown legs are densely covered with long and short hairs; a pale gray oval abdomen is sparsely covered with long brown hairs and densely covered with short light-brown hairs; no distinct patterns are observed on the abdomen. The female carapace and chelicerae are similar to those of the male; the legs are thicker and shorter than those of the male and some regions of the legs show no hair longitudinally; the abdomen is oval with thin light yellow hairs and without any distinct patterns. The spider has a body length of 3–6 cm or 6–10 cm with the legs extended (Figure 1B). The average weight of a mature spider is 4.11 ± 0.85 g (female) or 3.28 ± 0.67 g (male). It lives underground on open areas of hillsides or at the fringe of cultivated lands, but rarely in regions of dense forest or heavy undergrowth. The burrow in which the spider lives is constructed horizontally or with a slight slant and is usually 6–13 cm wide and 60–70 cm long. The entrance of the burrow is often covered with white spider silk, and the entire burrow appears as a loose webbing tube covered with silk around the inner wall (Figure 1C,D).

Spider venoms are known to contain several classes of peptide toxins that target voltage-gated ion channels and have been considered as a potential source of new compounds with specific pharmacological properties [2,6,7]. The potential of venom components as pharmacological tools and as potential leads for the development of new drugs and pesticides has recently been recognized. As a result, venoms and toxins have generated broad interest in the scientific community and in the agrochemical and pharmaceutical industries in recent years. Moreover, different spider species contain distinct toxin molecules. The venom of the spider *S*. *jiafu* could be a novel source for the identification of novel peptide toxins acting on voltage-gated ion channels. Therefore, in the present study, we conducted a biochemical and electrophysiological investigation of the venom of *S*. *jiafu*. We showed that the venom contains diverse peptides and possesses inhibitory activities on voltage-gated Na^+^, K^+^ and Ca^2+^ channels in rat dorsal root ganglion (DRG) neurons. These results provide clues for the purification and characterization of specific toxins in future studies.

## 2. Results and Discussion

### 2.1. Reversed-Phase High-Performance Liquid Chromatography (RP-HPLC), Matrix-Assisted Laser-Desorption/Ionization Time-of-Flight Mass Spectrometry (MALDI-TOF-MS) and Sodium Dodecyl Sulfate-Polyacrylamide Gel Electrophoresis (SDS-PAGE) Analyses of the Venom

**Figure 2:**
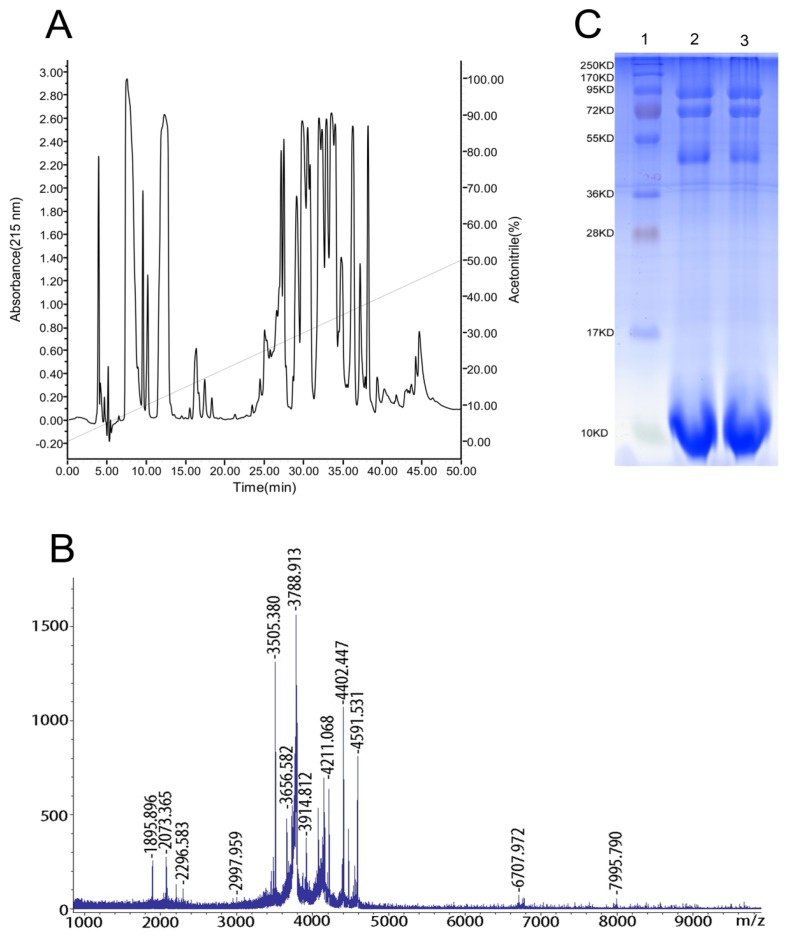
The complexity of the venom peptides: (**A**) reversed-phase high-performance liquid chromatography (RP-HPLC) separation of 1 mg of soluble venom from *S*. *jiafu* in an analytical C18 column equilibrated with solution A (distilled water in 0.1% TFA), using a gradient from 0% to 50% of solution B (acetonitrile in 0.1% TFA) over 50 min with a flow rate of 1 mL/min. Absorbance was read at 215 nm. (**B**) Matrix-assisted laser-desorption/ionization time-of-flight mass spectrometry(MALDI-TOF-MS) of *S*. *jiafu* venom. (**C**) Sodium dodecyl sulfate-polyacrylamide gel electrophoresis (SDS-PAGE) of the venom of the spider *S*. *jiafu*. 1: Marker; 2 and 3: Venom.

The spider *S*. *jiafu* can expel venom from the chelicerae after electrical stimulation. The venom is a clear and colorless liquid, easily soluble in water. Each *S*. *jiafu* spider yields approximately 5–20 μL venom. A typical RP-HPLC chromatogram of the venom of the spider *S*. *jiafu* is shown in Figure 2A. More than 40 fractions were eluted and monitored at 215 nm. Most of the fractions were eluted with retention times of 5–15 min and 25–40 min, corresponding to 5%–15% and 25%–40% acetonitrile, respectively. The fractions eluted at 5–15 min did not display any mass peaks in MALDI-TOF-MS spectra, indicating that they may contain low molecular mass organic compounds and salts. Spider venoms are commonly composed of proteins, peptides, low molecular mass organic compounds and salts, and peptides are the most abundant components of most spider venoms [8]. Therefore, we determined the mass distribution of the venom peptides by MALDI-TOF MS. As shown in Figure 2B, approximately 20 peaks were detected in a mass range from 1000 Da to 10,000 Da. Most of these peaks were localized in the range of 3000–4500 Da.

To explore the molecular weight distribution of the venom proteins, the venom was analyzed by SDS-PAGE using standard protocols. Except for polypeptides with molecular weights below 10 kD, the venomous proteins were mainly distributed in three bands on SDS-PAGE corresponding to the molecular weights of approximately 50 kD, 72 kD and 90 kD (Figure 2C).

**Figure 3:**
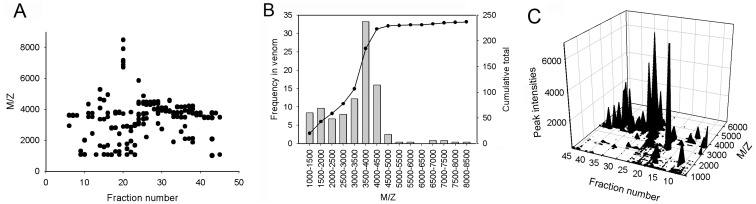
(**A**) Distribution of peptide molecular masses as a function of RP-HPLC elution time as determined by MALDI-TOF-MS analysis of individual RP-HPLC fractions of the venom from *S*. *jiafu*. (**B**) Histograms show the abundance of peptide toxins in the venom, sorted into 500 Da molecular mass classes. The overlaid curve shows the cumulative total of peptide masses identified from LC-MALDI analyses. (**C**) 3D venom landscape of *S*. *jiafu*. MALDI-TOF-MS peak intensities (*z*-axis, counts) were plotted as a function of hydrophobicity (*x*-axis, RP-HPLC fraction number) and mass (*y*-axis, *m*/*z*) to produce a 3D contour plot.

To examine the diversity of *S*. *jiafu* venomous peptides, MALDI-TOF-MS coupled offline to chromatographic separation was used to analyze the venom of the spider *S*. *jiafu*. This strategy was developed by Escoubas* et al.* [9] who plotted 3D landscapes to conceptualize the complexity of Australian funnel-web spider venoms. Their studies demonstrated that the venoms from these spiders contain many hundreds of peptides that follow a bimodal distribution, with the majority of peptides in the 3000–5000 Da mass range and a second less pronounced group in the 6500–8500 Da range. Similar results were reported in our previous studies of venoms from the Chinese tarantula spiders *Ornithoctonus huwena* [10], *Ornithoctonus hainana* [11] and *Chilobrachys jingzhao* [12]. We focused exclusively on the peptide fractions in the mass range from 1000 Da to 10,000 Da. As shown in Figure 3A, peptides of several masses were detected in almost all fractions analyzed, and some fractions even contained more than 10 peptides with different masses, suggesting that multiple peptides were co-eluted in each fraction. More than 200 peptides of different masses were identified, highlighting the complexity of the peptides in the venom (Figure 3B).

All the experimental data were integrated to construct a 3D venom landscape in which the mass spectral intensities were plotted as a function of both *m*/*z* and the RP-HPLC elution time (Figure 3C), thus providing a detailed overview of *S*. *jiafu* venomous peptides. Different peptides that were not completely separated by RP-HPLC can be distinguished by their relative mass values in the 3D plot.

### 2.2. Effects of the Venom on Voltage-Gated Sodium Channels (VGSCs)

VGSCs (Na_v_) are essential for the rapid depolarization of nerve and muscle. Na_v_ channels initiate and propagate action potentials in excitable cells. Because they underlie several clinical disorders such as epileptic seizures and cardiac arrhythmias, they are important drug targets [13]. Two types of Na^+ ^currents (I_Na_) have been identified on the basis of their sensitivity to tetrodotoxin (TTX), one is insensitive to TTX (up to 0.1 mM) while the other type is blocked by approximately 1 nM TTX [14]. Both types of Na_v_ channels are present in rat DRG neurons.

**Figure 4:**
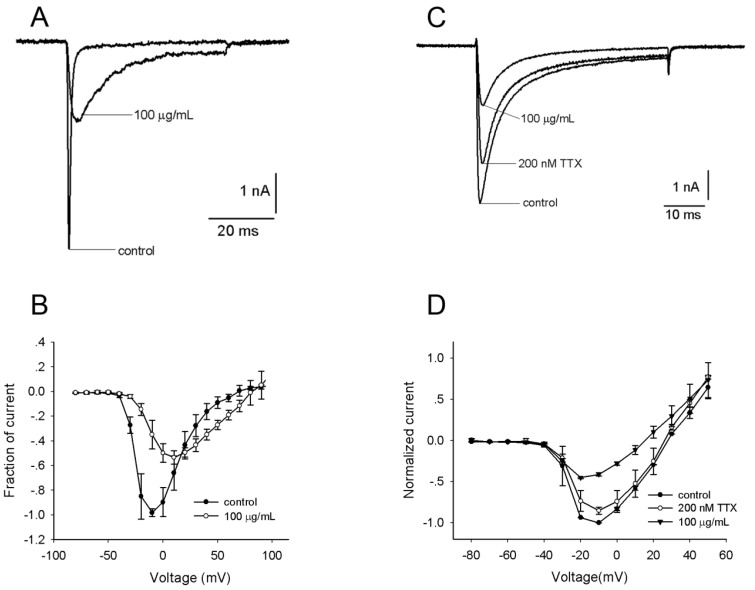
Effects of *S*. *jiafu* venom on whole-cell Na_v_ channel currents in rat dorsal root ganglion (DRG) neurons. (**A**) Tetrodotoxin-sensitive (TTX-S) Na^+^ currents were induced by a 50-ms depolarized potential of −10 mV from a holding potential of −80 mV in large DRG neurons. TTX-S current amplitude was blocked to 64.0% ± 6.2% (*n =* 5) by 100 μg/mLvenom. (**B**) Current-voltage (I-V) curves of TTX-S. For current-voltage curves, the currents were elicited by a series of 50-ms depolarizations from a holding potential of −80 mV, with a test potential ranging from −80 mV to +90 mV at increments of +10 mV. (**C**) Na^+^ currents were evoked by a 50-ms depolarized potential of −10 mV from a holding potential of −80 mV in medium size DRG neurons. At 200 nM tetrodotoxin (TTX), the remnant tetrodotoxin-resistant (TTX-R) currents were inhibited by 46.5% ± 3.8% (*n =* 5) in the presence of 100 μg/mL venom. (**D**) Current-voltage (I-V) curves of TTX-R. For current-voltage curves, the currents were elicited by a series of 50-ms depolarizations from a holding potential of −80 mV, with a test potential ranging from −80 mV to +50 mV at increments of +10 mV. The data were expressed as mean ± SE (*n =* 5).

The effects of the *S*. *jiafu* venom on Na_v_ channels in rat DRG neurons were therefore examined using the whole-cell patch-clamp technique. DRG neurons with a large diameter (>35 μm) and those with a relatively small diameter (<20 μm) were selected for measuring tetrodotoxin-sensitive (TTX-S) and tetrodotoxin-resistant (TTX-R) Na^+^ currents, respectively [15]. The venom had inhibitory activity against Na_v_ channels in rat DRG neurons. As shown in Figure 4, 100 μg/mL venom could not only suppress 64.0% ± 6.2% (*n =* 5) of TTX-S currents but also delay channel inactivation (Figure 4A).

The time constants for inactivation before and after the treatment of the venom were 0.7 ± 0.2 ms and 7.5 ± 1.4 ms (*n =* 5), respectively. In the presence of the venom, the remnant currents could be completely blocked by 200 nM TTX, indicating the TTX-S property of the VGSC (Na_v_) currents expressed in large diameter neurons. The current-voltage (I-V) curves showed that the venom changed the activation of TTX-S currents by a depolarizing shift of the activation voltage of the peak current from −10 mV to +10 mV (*n =* 5) (Figure 4B). The half-activation voltage in the presence of the venom was −8.9 ± 0.2 mV, compared to −24.6 ± 0.1 mV (*n =* 5) in the control. To test the inhibitory effect of the venom on TTX-R currents, 200 nM TTX was used to separate the TTX-R currents from the TTX-S currents. As shown in Figure 4C, the amplitude of the remnant TTX-R currents was reduced by 46.5% ± 3.8% (*n =* 5) in the presence of 100 μg/mL venom, suggesting that antagonists of TTX-R Na_v_ channels are present in the venom. The current-voltage (I-V) curves showed that the venom changed the activation of TTX-R currents by a depolarizing shift of the activation voltage of the peak current from −10 mV to −20 mV (*n =* 5) (Figure 4D). The half-activation voltages before and after the venom treatment were −22.7 ± 0.5 mV to −27.2 ± 0.2 mV (*n =* 5), respectively. Rat DRG neurons express two isoforms of TTX-R Na_v_ channels, Na_v_1.8 and Na_v_1.9. Na_v_1.8 is involved in neuropathic pain [16], whereas Na_v_1.9 is implicated in inflammatory pain modulation through the upregulation of neuronal excitability in DRG neurons [17]. Our data indicate that Na_v_1.8 and/or Na_v_1.9 antagonists can be identified in the venom of *S*. *jiafu*.

### 2.3. Effects of the Venom on Voltage-Gated Potassium Channels

**Figure 5:**
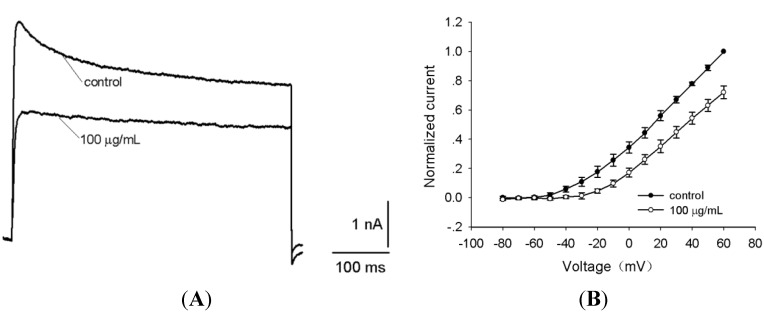
Effects of *S*. *jiafu* venom on whole-cell K_v_ channel currents in rat DRG neurons. (**A**) K_v_ currents were evoked by a 500-ms depolarized potential of 10 mV from a holding potential of −80 mV. Application of 100 μg/mL of venom led to a reduction of K^+ ^currents of 44.2% ± 5.3% (*n =* 5); (**B**) Normalized current-voltage relation of K^+ ^currents before (filled circles) and after (open circles) venom treatment. Currents were elicited by a series of 500-ms depolarizations from a holding potential of −80 mV to +60 mV in 10-mV steps. The data were expressed as mean ± S.E (*n =* 5).

DRG neurons express many types of K^+^ channels including voltage-gated (K_v_), inwardly rectifying (K_ir_), Ca^2+ ^activated (K_Ca_) and background (leak, K_2P_) K^+ ^channels. These channels contribute to the regulation of membrane repolarization, resting membrane potential, frequency of firing, and excitability of sensory neurons [18,19]. Among various K^+^ channels, K_v_ channels play a crucial role in returning the depolarized cell to the resting state, and the inhibition of these channels leads to the broadening of action potentials [20]. Our study showed that 100 μg/mL venom inhibited K^+^ currents by approximately 44.2% ± 5.3% (*n =* 5) (Figure 5A). Figure 5B shows the current-voltage curves obtained before and after venom treatment, indicating that the venom altered the initial activation voltage of K^+^ currents in rat DRG neurons. At a concentration of 100 μg/mL, it shifted the initial activation voltage from −50 ± 2.6 mV to −20 ± 1.7 mV. The half-activation voltages before and after the application of the venom were 21.9 ± 2.8 mV and 25.5 ± 2.0 mV, respectively.

### 2.4. Effects of the Venom on Voltage-Gated Calcium Channels

**Figure 6:**
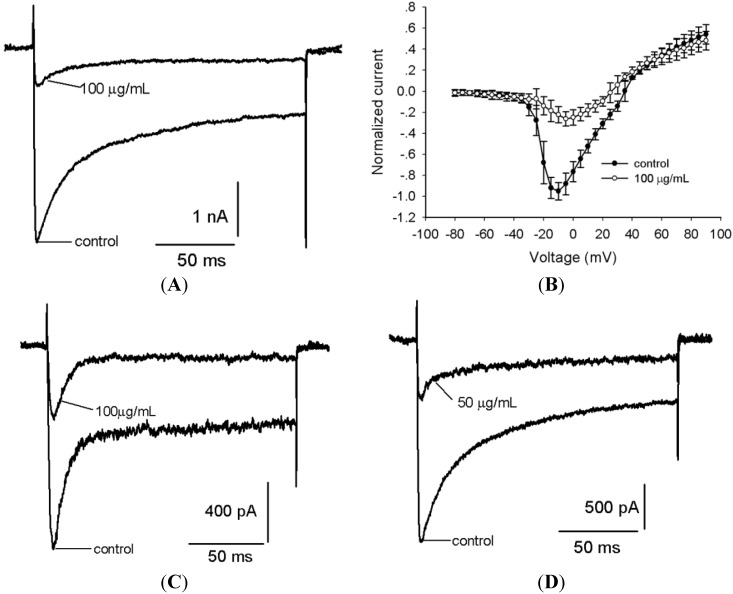
Effects of *S*. *jiafu* venom on whole-cell Ca_v_ channel currents in rat DRG neurons. (**A**) The application of 100 μg/mL of venom inhibited 65.4% ± 9.5% (*n =* 5) of total Ca_v _currents evoked by a 150-ms depolarization to 10 mV from a holding potential of −90 mV. (**B**) Normalized current-voltage relationships of Ca_v_ currents in the presence and absence of 100 μg/mL of venom. Currents were elicited by a series of 150-ms depolarizations from −80 mV to +90 mV in 5-mV steps with a holding potential of −90 mV. The data were expressed as mean ± SE (*n =* 5). (**C**) The application of 100 μg/mL of venom inhibited 77.7% ± 9.9% (*n =* 5) of LVA-Ca_v_ currents evoked by a 150-ms depolarization to −20 mV from a holding potential of −90 mV. (**D**) The application of 50 μg/mL of venom inhibited 74.9% ± 2.8% (*n =* 5) of HVA-Ca_v _currents elicited by a 150-ms depolarization to +10 mV from a holding potential of −40 mV.

Ca^2^^+^ entry into cells through voltage-gated Ca^2^^+^ channels mediates many physiological processes including neurotransmitter release, neurosecretion, neuronal excitation, survival of neurons, and regulation of gene expression. Currently, five main types of Ca^2+^ channels (T, L, N, P/Q, and R) have been identified in vertebrate cells based on their physiological and pharmacological properties [21,22,23]. High-voltage-activated (HVA) L-, N-, P/Q- and R-type Ca^2+ ^channels require strong depolarization for activation and are essentially implicated in neurotransmitter release. Low voltage-activated (LVA) T-type currents can be activated by small depolarizations, are rapidly inactivated, and modulate pacemaker type repetitive activity in cardiac cells and neurons [24]. As shown in Figure 6A, the addition of 100 μg/mL venom could inhibit approximately 65.4% ± 9.5% (*n =* 5) of total Ca_v_ currents in rat DRG neurons, which were activated by a 150-ms step depolarization to +10 mV from a holding potential of −90 mV. Figure 6B shows the I-V curves of total Ca^2+ ^currents, indicating that the venom caused no change on the initial activated voltage, the active voltage of peak inward current and the reversal potential of Ca^2+ ^currents.

Next, we investigated whether the venom had inhibitory activity against both types of Ca_v_ channels in rat DRG neurons. As shown in Figure 6C, the application of 100 μg/mL venom led to an approximately 77.7% ± 9.9% (*n =* 5) reduction of LVA currents elicited by a 150-ms step depolarization to −40 mV from a holding potential of −90 mV. T-type channels are widely distributed in pain pathway neurons and implicated in pain modulation. Since no peptide antagonist of T-type channels has been identified to date, the identification of inhibitors of T-type channels from the venom of *S*. *jiafu* is important. Meanwhile, treatment with 50 μg/mL venom resulted in a 74.9% ± 2.8% (*n =* 5) reduction of HVA currents activated by a 150 ms step depolarization to +10 mV from a holding potential of −40 mV (Figure 6D). Several types of HVA Ca_v _channels are present in rat DRG neurons. N-type channel blockers profoundly attenuate hyperalgesia and allodynia in response to mechanical, chemical or thermal stimulation [25]. P/Q-type channels have been linked genetically to specific diseases including epilepsy, ataxia and migraines [26]. We expect that inhibitors of a specific HVA Ca_v_ channel would be identified from the venom of *S*. *jiafu*.

### 2.5. Bioactivity Assays

We first examined the toxic effects of the venom against mice. It was found that no visible symptom or behavioral change was detected after intraperitoneal injection of the venom into mice even at doses less than 10 mg/kg body weight; at a dose of 20 mg/kg body weight, mice showed mild symptoms of poisoning including a reduction of the level of activity, rapid breathing, and abdominal trembling. These symptoms subsided within 30 min; at a dose of 46.30 mg/kg body weight, mice exhibited obvious symptoms of poisoning and finally died 24 min after venom injection. We next checked toxic effects of the venom on the American cockroach (*Periplaneta Americana*). A large dose injection produced symptoms of poisoning including hind limb paralysis, stumbling during crawling, foot shaking and even death. The LD_50 _of *S*. *jiafu* venom in the cockroach was 84.24 μg/g body weight, which is approximately 25% of the LD_50 _of *S*. *huwena* venom in the cockroach (the LD_50_ of *S*. *huwena* crude extract against cockroaches is 300 μg/g body weight) [27]. Our data indicate that the venom might contain some factors that target the ion channels of insects and could therefore be valuable for the development of novel insecticides. Further studies will be needed to identify and characterize the components acting on insect ion channels.

## 3. Experimental Section

### 3.1. Venom Collection

Thespiders were collected from the hilly area of Ninming county of Guangxi Province in the south of China. Adult female *S*. *jiafu* spiders were maintained in wooden boxes or plastic pails covered with plastic netting and given water daily. The spiders were fed pig liver (cut into pieces of 1–2 cm^3^), cockroaches or worms every seven days. Stimulation of female *S. jiafu* every two weeks as described in our previous work [28]. The venom was collected by using an electro-pulse stimulator. The two output electrodes of the stimulator were placed in contact with the sides of the root of the spider’s chelicerae. Physiological saline was used to enhance electrical conduction. Electrostimulation at 10–15 V and 25–80 Hz with a pulse time of 0.7 ms was applied across the chelicerae. Expelled venom was collected from the fang tips with a tube, pooled and freeze-dried. The freeze-dried crude venom was stored at −20 °C prior to analysis.

### 3.2. RP-HPLC Analysis

The venom powder was dissolved with water to a final concentration of 10 mg/mL, centrifuged at 14,000 rpm for 30 min at 4°C and filtered using a 0.22 μm microfilter (MILLEX GP, Millipore Ireland Ltd., Cork, Ireland). The venom solution was analyzed by RP-HPLC on a C18 column (phenomenex 100 Å, 4.6 mm × 250 mm, 5 μm) using a Waters Alliance 2695 (Milford, MA, USA). Venom components were eluted using a linear acetonitrile gradient (0%–50% acetonitrile/0.1% TFA in 50 min) at a flow rate of 1.0 mL/min [29]. Fractions (1 mL/tube) were collected manually by monitoring the absorbance at 215 nm. Each fraction was lyophilized and reconstituted in 30 μL of 50% acetonitrile in water/0.1% TFA prior to MALDI-TOF-MS analysis.

### 3.3. MALDI-TOF-MS Analysis

The venom was desalted with a ZIP-TIP and analyzed using MALDI-TOF-MS (Ultraflex, Bruker Daltonics, Bremen, Germany). Ionization was achieved by irradiation with a nitrogen laser (337 nm) with a 20 kV acceleration voltage. 10 mg/mL α-cyano-4 hydroxy-cinnamic acid (CCA) was used as the matrix. To analyze the fractions obtained by RP-HPLC, 1 μL of each fraction was applied to a well overlaid with 1 μL of CCA. Data were acquired using Bruker Daltonics Flexcontrol software (Bremen, Germany) and exported with Flex analysis software (Bremen, Germany). All analyses were conducted in a reflector mode. 

### 3.4. SDS-PAGE Analysis

The venom powder was dissolved with water to a final concentration of 10 mg/mL, centrifuged at 14,000 rpm for 30 min at 4 °C and filtered using a 0.22 μm microfilter (MILLEX GP, Millipore). The venom solution was analyzed by SDS-PAGE (15% separation gel and 5% stacking gel) using standard protocols to check the MW range and stained with Coomassie blue G-250 (Sigma-Aldrich, St. Louis, MO, USA).

### 3.5. Electrophysiological Test

Whole-cell voltage-clamp recordings of voltage-gated ion currents were made in rat DRG neurons which were acutely dissociated from 30-day old Sprague-Dawley rats and maintained in short-term primary culture according to the method described by Xiao and Liang [30]. DRG neurons with a large diameter (>35 μm) and those with a relatively small diameter (<20 μm) were selected for measuring TTX-S and TTX-R Na^+^ currents, respectively. Meanwhile, TTX (final concentration: 200 nM) was used to separate TTX-R Na^+^ currents from TTX-S Na^+^ currents. Electrophysiological recordings were performed at room temperature. Suction pipettes (2.0–4.0 MΩ) were made from borosilicate glass capillary tubes using a two-step pulling procedure on a vertical micropipette puller. For Na^+^ current recording, the pipette solution contained (in mM) CsCl 145, MgCl_2_·6H_2_O 4, hydroxyethyl piperazine ethane-sulfonic acid (HEPES) 10, ethylene glycol tetraacetate (EGTA) 10, glucose 10, adenosine-triphosphate (ATP) 2 (pH 7.2); and the external solution contained (in mM) NaCl 145, KCl 2.5, CaCl_2_ 1.5, MgCl_2_·6H_2_O 1.2, HEPES 10, and d-glucose 10 (pH 7.4). For K^+^ current recording, the pipette solution contained (in mM) KCl 135, KF 25, NaCl 9, CaCl_2_ 0.1, MgCl_2_ 1, EGTA 1, HEPES 10 and ATP-Na_2_ 3, adjusted to pH 7.4 with 1 M KOH; and the external bath solution contained (in mM) NaCl 150, KCl 30, CaCl_2_ 5, MgCl_2_ 4, TTX 0.3, HEPES 10 and d-glucose 10, adjusted to pH 7.4 with 1 M NaOH. For Ca^2+^ current recording, the internal solution contained (in mM) Cs-methane sulfonate 110, phosphocreatine 14, HEPES 10, EGTA 10, ATP-Mg 5, adjusted to pH 7.3 with CsOH; the external solution contained (in mM) BaCl_2 _ 10, tetraethylammonium (TEA)-Cl 125, TTX 0.3 and HEPES 10, adjusted to pH 7.4 with TEA-OH. To eliminate the potential influence of differences in osmotic pressure, all internal and external solutions were adjusted to 280 ± 5 mOsm with sucrose. Experimental data were collected and analyzed with Pulse/Pulsefit 8.0 (HEKA Electronics, Lambrecht, Germany) and Sigmaplot 10.0 (Systat Software, San Jose, CA, USA). Macroscopic currents were filtered at 10 kHz, digitized at 3 kHz with an EPC-10 patch-clamp amplifier (HEKA Electronics). Series resistance was kept near 5 MΩ and compensated 65%–70%. Linear capacitive and leakage currents were digitally subtracted using the P/4 protocol. All data are presented as means ± S.E., and *n* is the number of independent experiments.

### 3.6. Bioactivity Assays

The toxicity of the venom of *S*. *jiafu* was qualitatively assayed by intraperitoneal injection into 18–20 g mice of both sexes at doses of 1–10 mg/kg using 100 μL solutions [in 0.9% (w/v) normal saline] and intra-abdominal injection into adult male cockroaches (*Periplaneta* *americana*) with body weights of 0.8–1.2 g at doses of 150–500 μg/g using 10 μL solutions [in 0.85% (w/v) normal saline]. The LD_50_ was determined using five doses per animal in six animals. Mice and cockroaches were monitored for 24 h after injection. Data were analyzed using the modified Kaber method.

## 4. Conclusions

Spider venoms represent a valuable source of biologically active substances that selectively target a variety of vital physiological functions in both insects and mammals. The venom of *S*. *jiafu* is novel spider venom that has remained unexplored until now. In the present study, we performed a preliminary analysis of the biochemical and pharmacological properties of this venom. Our results indicate that the venom of *S*. *jiafu* contains a complex mixture of peptides, as revealed by the venom landscape analysis. The molecular masses of the venom peptides range from 1000 Da to 10,000 Da, with most peptides in the mass range of 3000–4500 Da. The venom contains neurotoxins with activity against voltage-gated Na^+^, K^+^ and Ca^2+^ channels in rat DRG neurons. Further analysis should identify antagonists of TTX-R Na_v_ channels and T-type Ca_v_ channels, as the venom showed inhibitory activities against both channels. The results of the present study provide a basis for further case-by-case investigations of peptide toxins from this venom.
